# Supplementary material for: High incidence of plasmid-mediated quinolone resistance genes among ciprofloxacin-resistant clinical isolates of Enterobacteriaceae at a tertiary care hospital in Puducherry, India
Source: PeerJ. 2016 May 5;4:e1995. doi: 10.7717/peerj.1995 (PMC4860338; doi:10.7717/peerj.1995)
Supplement: Data S1 [file peerj-04-1995-s001.docx]

| **Annual Fluoroquinolone Prescribing Data** | | | | | | | | | | | | | | | | |
| --- | --- | --- | --- | --- | --- | --- | --- | --- | --- | --- | --- | --- | --- | --- | --- | --- |
| MONTH | Year | | | | | | | | | | | | | | | |
|  | 2012 | | | | 2013 | | |  | 2014 | | |  | 2015 | | |  |
|  | Ciprofloxacin Tablets  (500 mg) | Levofloxacin Tablets  (500 mg) | Ciprofloxacin IV  (200mg/100mL) | Ofloxacin IV  (200mg/100mL) | Ciprofloxacin Tablets  (500 mg) | Levofloxacin Tablets  (500 mg) | Ciprofloxacin IV  (200mg/100mL) | Ofloxacin IV  (200mg/100mL) | Ciprofloxacin Tablets  (500 mg) | Levofloxacin Tablets  (500 mg) | Ciprofloxacin IV  (200mg/100mL) | Ofloxacin IV  (200mg/100mL) | Ciprofloxacin Tablets  (500 mg) | Levofloxacin Tablets  (500 mg) | Ciprofloxacin IV  (200mg/100mL) | Ofloxacin IV  (200mg/100mL) |
| January | 22920 | NA | 1970 | NA | 34070 | NA | 710 | NA | 29510 | 1183 | 1282 | 52 | 39580 | NA | 1089 | 60 |
| February | 26870 |  | 1210 |  | 28340 |  | 777 |  | 23860 | 953 | 1395 | 64 | 23740 |  | 1515 | 50 |
| March | 21070 |  | 995 |  | 26230 |  | 871 |  | 15710 | 1054 | 1665 | 30 | 25918 |  | 1455 | 65 |
| April | 22960 |  | 780 |  | 29050 |  | 791 |  | 28270 | 830 | 1055 | 50 | 34570 |  | 1323 | 67 |
| May | 28690 |  | 3215 |  | 25870 |  | 1451 |  | 25950 | 956 | 900 | 75 | 33221 |  | 1570 | 71 |
| June | 23660 |  | 1355 |  | 11670 |  | 1358 |  | 12220 | 740 | 1725 | 51 | 29831 |  | 1321 | 59 |
| July | 44120 |  | 1245 |  | 26600 |  | 1691 |  | 31570 | 460 | 1153 | 47 | 23471 |  | 1229 | 43 |
| August | 32340 |  | 1380 |  | 24780 |  | 2450 |  | 9930 | Out of Stock | 1770 | 53 | 36270 |  | 1340 | 72 |
| September | 30510 |  | 1470 |  | 30740 |  | 1560 |  | 34590 | 1510 | 1280 | 71 | 33216 |  | 819 | 89 |
| October | 14770 |  | 1285 |  | 22720 |  | 1390 |  | 26620 | 1130 | 1288 | 112 | 27890 |  | 721 | 53 |
| November | 21480 |  | 1743 |  | 23570 |  | 981 |  | 18020 | 900 | 1453 | 87 | 23690 |  | 1081 | 67 |
| December | 27290 |  | 1546 |  | 23280 |  | 1013 |  | 26690 | 1030 | 2345 | 75 | 24519 |  | 1321 |  |
