# Supplementary material for: High incidence of plasmid-mediated quinolone resistance genes among ciprofloxacin-resistant clinical isolates of Enterobacteriaceae at a tertiary care hospital in Puducherry, India
Source: PeerJ. 2016 May 5;4:e1995. doi: 10.7717/peerj.1995 (PMC4860338; doi:10.7717/peerj.1995)
Supplement: Data S2 [file peerj-04-1995-s002.doc]

| **Phylogeny of *aac(6')-Ib-cr* nucleotide sequences identified in this study** | | | | | | | | | | | | | | | | | | | | | | | | |
| --- | --- | --- | --- | --- | --- | --- | --- | --- | --- | --- | --- | --- | --- | --- | --- | --- | --- | --- | --- | --- | --- | --- | --- | --- |
| **Clinical Isolates** | **1793** | **1871** | **RE0707** | **3101** | **007** | **678** | **1017** | **1030** | **1077** | **1115** | **1116** | **1729** | **1758** | **1966** | **2674** | **2839** | **2858** | **2871** | **2883** | **2995** | **3148** | **10727** | **11590** | **171040** |
| **1793** |  |  |  |  |  |  |  |  |  |  |  |  |  |  |  |  |  |  |  |  |  |  |  |  |
| **1871** | **3.603** |  |  |  |  |  |  |  |  |  |  |  |  |  |  |  |  |  |  |  |  |  |  |  |
| **RE0707** | **4.756** | **2.874** |  |  |  |  |  |  |  |  |  |  |  |  |  |  |  |  |  |  |  |  |  |  |
| **3101** | **1.129** | **2.046** | **3.368** |  |  |  |  |  |  |  |  |  |  |  |  |  |  |  |  |  |  |  |  |  |
| **007** | **3.783** | **1.726** | **0.285** | **3.095** |  |  |  |  |  |  |  |  |  |  |  |  |  |  |  |  |  |  |  |  |
| **678** | **2.093** | **3.419** | **2.274** | **4.746** | **2.310** |  |  |  |  |  |  |  |  |  |  |  |  |  |  |  |  |  |  |  |
| **1017** | **2.699** | **2.222** | **2.184** | **2.586** | **1.735** | **2.940** |  |  |  |  |  |  |  |  |  |  |  |  |  |  |  |  |  |  |
| **1030** | **4.782** | **0.246** | **1.565** | **2.351** | **2.435** | **3.448** | **3.881** |  |  |  |  |  |  |  |  |  |  |  |  |  |  |  |  |  |
| **1077** | **2.792** | **3.288** | **3.582** | **2.433** | **3.267** | **2.917** | **3.342** | **3.050** |  |  |  |  |  |  |  |  |  |  |  |  |  |  |  |  |
| **1115** | **3.838** | **1.928** | **1.526** | **4.760** | **2.648** | **3.312** | **2.252** | **1.227** | **2.271** |  |  |  |  |  |  |  |  |  |  |  |  |  |  |  |
| **1116** | **0.279** | **3.797** | **3.790** | **2.334** | **3.901** | **2.118** | **2.685** | **3.591** | **3.275** | **3.208** |  |  |  |  |  |  |  |  |  |  |  |  |  |  |
| **1729** | **2.473** | **1.958** | **2.053** | **2.292** | **2.265** | **2.998** | **2.413** | **3.270** | **3.775** | **2.847** | **2.461** |  |  |  |  |  |  |  |  |  |  |  |  |  |
| **1758** | **2.688** | **2.653** | **2.846** | **2.468** | **2.862** | **2.890** | **1.749** | **2.979** | **2.818** | **3.818** | **1.615** | **2.256** |  |  |  |  |  |  |  |  |  |  |  |  |
| **1966** | **3.080** | **2.493** | **3.615** | **2.818** | **4.833** | **1.686** | **2.603** | **2.004** | **2.536** | **2.720** | **2.641** | **3.804** | **2.906** |  |  |  |  |  |  |  |  |  |  |  |
| **2674** | **2.663** | **1.745** | **3.344** | **2.146** | **1.619** | **2.803** | **1.779** | **3.152** | **2.720** | **2.451** | **3.032** | **3.821** | **3.588** | **2.310** |  |  |  |  |  |  |  |  |  |  |
| **2839** | **3.434** | **2.494** | **2.335** | **2.685** | **2.566** | **3.882** | **2.401** | **2.971** | **4.907** | **2.377** | **3.626** | **1.237** | **2.816** | **2.583** | **2.674** |  |  |  |  |  |  |  |  |  |
| **2858** | **4.751** | **0.058** | **2.792** | **1.868** | **1.739** | **3.504** | **1.984** | **0.266** | **2.979** | **1.640** | **4.748** | **2.125** | **3.387** | **2.015** | **1.660** | **2.281** |  |  |  |  |  |  |  |  |
| **2871** | **2.206** | **2.219** | **3.366** | **0.231** | **3.730** | **3.662** | **3.179** | **1.760** | **2.831** | **3.741** | **1.798** | **1.486** | **2.777** | **2.052** | **2.423** | **1.608** | **1.838** |  |  |  |  |  |  |  |
| **2883** | **2.359** | **1.615** | **2.412** | **3.696** | **1.150** | **0.272** | **1.448** | **3.432** | **2.494** | **2.186** | **2.224** | **2.483** | **2.429** | **3.555** | **1.571** | **3.636** | **1.402** | **3.845** |  |  |  |  |  |  |
| **2995** | **2.298** | **1.724** | **3.736** | **1.739** | **2.172** | **4.652** | **1.619** | **2.633** | **2.678** | **3.055** | **2.590** | **3.386** | **2.342** | **2.590** | **1.336** | **2.958** | **1.824** | **1.907** | **2.399** |  |  |  |  |  |
| **3148** | **2.045** | **2.591** | **3.427** | **0.243** | **3.043** | **3.679** | **3.516** | **2.348** | **3.119** | **4.786** | **2.193** | **3.046** | **2.902** | **3.036** | **2.410** | **3.026** | **2.794** | **0.311** | **4.740** | **1.691** |  |  |  |  |
| **10727** | **1.498** | **2.742** | **3.768** | **1.235** | **3.264** | **3.629** | **2.401** | **2.846** | **2.153** | **3.244** | **2.518** | **3.302** | **1.991** | **2.545** | **2.242** | **2.956** | **2.883** | **2.003** | **3.839** | **0.146** | **1.881** |  |  |  |
| **11590** | **3.222** | **2.519** | **2.355** | **2.246** | **2.287** | **4.734** | **2.364** | **3.184** | **4.831** | **2.601** | **3.554** | **1.295** | **2.512** | **2.641** | **2.702** | **0.035** | **2.402** | **1.585** | **3.659** | **2.876** | **3.155** | **2.672** |  |  |
| **171040** | **3.405** | **2.380** | **2.323** | **2.685** | **2.566** | **4.709** | **2.521** | **2.958** | **4.932** | **2.532** | **3.491** | **1.218** | **2.769** | **2.749** | **2.853** | **0.031** | **2.648** | **1.691** | **3.825** | **3.028** | **2.916** | **2.782** | **0.044** |  |
| **A931** | **2.874** | **2.382** | **3.111** | **2.963** | **4.796** | **1.978** | **2.761** | **2.391** | **2.367** | **2.454** | **2.941** | **3.736** | **2.844** | **0.086** | **2.358** | **2.667** | **2.286** | **2.998** | **3.628** | **2.820** | **2.777** | **2.470** | **2.819** | **2.610** |

**±The values in the table are provided in the terms of percentages, i.e., the number of changes per 100 nucleotides of the *aac(6’)-Ib-cr* gene sequences.**
